# Transcriptome Analysis Revealed Inflammation Is Involved in the Impairment of Human Umbilical Vein Endothelial Cells Induced by Post-hemorrhagic Shock Mesenteric Lymph

**DOI:** 10.3389/fimmu.2020.01717

**Published:** 2020-09-09

**Authors:** Qi Wang, Zhen-Fen Chi, Di Wei, Zhen-Ao Zhao, Hong Zhang, Li-Min Zhang, Yan-Xu Liu, An-Ling Kang, Meng Zhao, Peng Wang, Ling-Hu Nie, Chun-Yu Niu, Zi-Gang Zhao

**Affiliations:** ^1^Institute of Microcirculation, Hebei North University, Zhangjiakou, China; ^2^Pathophysiology Experimental Teaching Center of Basic Medical College, Hebei North University, Zhangjiakou, China; ^3^Beijing Institute of Genomics, Chinese Academy of Sciences, Beijing, China; ^4^Basic Medical College, Hebei Medical University, Shijiazhuang, China; ^5^Key Laboratory of Critical Disease Mechanism and Intervention in Hebei Province, Shijiazhuang, China

**Keywords:** hemorrhagic shock, mesenteric lymph, endothelial cell, inflammation, C–C motif chemokine ligand 2

## Abstract

Vascular endothelial injury caused by post-hemorrhagic shock mesenteric lymph (PHSML) return is an important manifestation during refractory hemorrhagic shock. Using human umbilical vein endothelial cells (HUVECs) and transcriptome analysis, this study sought to investigate the molecular mechanism underlying the adverse effect of PHSML on vascular endothelium. Post-hemorrhagic shock mesenteric lymph was collected from male rats after they underwent hemorrhagic shock and following resuscitation, while normal mesenteric lymph (NML) was harvested from sham rats. Human umbilical vein endothelial cells were incubated with the culture medium containing either 10% phosphate buffered saline (Control), NML, or PHSML for 3 h, and then were harvested for RNA sequencing. In comparison with NML treated cells, 37 genes were differentially expressed in PHSML-treated HUVECs, including 32 upregulated genes and five downregulated genes. These differentially expressed genes were mainly enriched in inflammatory pathways, including signaling pathways for activation of the NOD-like receptors, NF-κB, and TNF. Furthermore, we found that C–C motif chemokine ligand 2 (CCL2) was increased significantly after PHSML treatment, and Bindarit, a CCL2 production inhibitor, attenuated the damage of HUVECs induced by PHSML. The results provide molecular evidence on vascular endothelium damage caused by PHSML. C–C motif chemokine ligand 2 might represent a new target for reducing vascular injury after severe hemorrhagic shock.

## Introduction

Ischemic hypoxia, hyper-inflammation, and oxidative stress induced by hemorrhagic shock lead to the injury of vascular endothelial cells, which is the main cause of aggravating microcirculation dysfunction and organ injury following refractory hemorrhagic shock. Numerous studies have shown that post-hemorrhagic shock mesenteric lymph (PHSML) return is the main cause of uncontrolled inflammation and acute distant organ damage (1–3). It is reported that PHSML can directly induce the injury of rat or human pulmonary microvascular endothelial cells and human umbilical vein endothelial cells (HUVECs), resulting in increased permeability of monolayer cells *in vitro* (4–6). However, precise molecular mechanisms have not been elucidated. Second generation sequencing technology is the most effective way to analyze the transcriptional profile (7). Through differential transcriptome analysis, one can study the pathophysiological mechanisms of cell injury and identify new targets for treatment and prevention of hemorrhagic shock. Human umbilical vein endothelial cell was mostly used to study vascular permeability and endothelial barrier function (8). In this study, we sought to explore the mechanism of PHSML injury to vascular endothelium, HUVECs were incubated with PHSML and the differential mRNA expression was investigated using RNA sequencing (RNA-seq) technology. Subsequently, differentially expressed genes were analyzed and verified using the bioinformatic and RT-PCR methods. As a representative gene among differentially expressed genes, C–C motif chemokine ligand 2 (CCL2) and its inhibitor, Bindarit, were used to further confirm the roles of these genes in mediating HUVEC injury.

## Materials and Methods

### Preparation of Mesenteric Lymph Samples

All experimental protocols involving animals were approved by the Laboratory Animal Research Committee of Hebei North University. Adult male Wistar rats (280–320 g) were purchased from the Experimental Animal Center of the Chinese Academy of Military Medical Sciences and were randomly divided into a sham group and a hemorrhagic shock group, with *n* = 12 for each group.

Animal models were established according to the routine method of a previous report (9). The rats received an inhalation anesthetic induction with isoflurane and a general anesthetization with 1% pentobarbital sodium (50 mg/kg, Merck, Germany). Then, bilateral inguinal region surgery was performed to separate the femoral artery and vein. Heparin sodium (1 ml/kg, 500 U/kg) was injected into the right femoral vein to prevent blood coagulation during the experiment. The right femoral artery was intubated using polyethylene tubing, which was connected to the PowerLab biological signal acquisition system (ADInstruments, Bella Vista NSW, Australia) for blood pressure monitoring. The left femoral vein was intubated and connected to the syringe for fluid resuscitation. The incision (about 4 cm) was cut along the abdominal white line. After the operation, the animals were kept stable for 10 min. The blood pressure was maintained at 40 ± 2 mmHg for 10 min by drawing blood using an automatic withdrawal-infusion machine (NE-1000, New Era Pump Systems Inc., Farmingdale, NY). The collected blood was mixed with an equal volume of Ringer’s solution and then slowly injected into the left femoral vein within 30 min for resuscitation. In the sham group, all the above operations were performed except bleeding or resuscitation, and the operation time was the same as that of the hemorrhagic shock model.

To prepare the lymph samples, we opened the abdomen along the incision, lifted the right intestine, and found the mesenteric lymphatic duct accompanying the superior mesenteric artery. The infusion needle was adjusted to a suitable bend and inserted into the mesenteric lymphatic duct. The lymph was then drained and extracted by a vacuum tube. After centrifugation at 850 *g* at 4°C for 10 min, the supernatant was collected at −80°C for cell experiment and ELISA analysis.

### Cell Culture and Treatment

Human umbilical vein endothelial cells (purchased from Cobioer Biosciences Co., Ltd, Nanjing, China, Catalog #: CBP60340) were cultured in BEGMTM (Bronchial Epithelial Cell Growth Medium; CC-3170, Lonza) containing 10% FBS at 37°C and 5% CO_2_. When the confluence of cells reached about 70%, the medium was changed into a culture medium containing 10% phosphate buffered saline (PBS), normal mesenteric lymph (NML), or PHSML. These cells were further cultured for 3 h for the RNA extraction.

### RNA Extraction, Library Construction, and Sequencing

Total RNA was extracted using Trizol Reagent, and the integrity of the RNA was analyzed by Agilent 2100 (Agilent Technologies, CA). A library was constructed using NEBNext^®^ UltraTM RNA Library Prep Kit for Illumina^®^ (New England Biolabs, Lspawich, MA, United States) according to instructions. RNA-seq was performed in Novogene Co., Ltd. The sequencing data description was shown in Table 1. Subsequently, TopHat2 software was used to analyze the data obtained with the reference genome. HTSeq v0.6.1 was used to analyze differentially expressed genes in different samples. Log2 (Fold Change) > 1 and *Q*-value < 0.005 were used as screening criteria (10). Fold Change represents the ratio of expression between two samples.

**TABLE 1:** Description of RNA-sequencing data.

**Sample name**	**Raw reads**	**Clean reads**	**Q20 (%)**	**Q30 (%)**	**Uniquely mapped**	**Multiple mapped**	**Exon mapped (%)**
Control	52370438	50765268	96.52	91.37	47,269,147 (93.11%)	1,019,113 (2.01%)	95.5
NML	50597550	49089518	96.51	91.35	45,597,648 (92.89%)	995,409 (2.03%)	95.4
PHSML	60822884	58448952	96.91	92.24	54,200,373 (92.73%)	1,192,927 (2.04%)	95.6

*HUVECs are treated by physiological saline (Control), normal mesenteric lymph (NML) and post-hemorrhagic shock mesenteric lymph (PHSML), respectively.*

### Functional Analysis of Differentially Expressed Genes

Gene Ontology (GO) enrichment of differentially expressed genes was analyzed by the GOseq R package with P-value less than 0.05 after the correction was considered to be significantly enriched (11). We used KOBAS v2.0 software to enrich and analyze the differentially expressed genes in Kyoto Encyclopedia of Genes and Genomes (KEGG) pathways.

### Quantitative Real-Time Polymerase Chain Reaction

Total RNA was extracted using Invitrogen TRIzol reagent (Thermo Fisher Scientific). After DNase, I digestion, an aliquot of total RNA (500 ng) was reverse transcribed with Takara PrimeScript RT Reagent Kit (RR037A, Takara). Real-time PCR was performed using a SYBR^®^ Premix Ex Taq^TM^ (DRR420A; Takara) on the Applied Biosystems StepOnePlus Real-Time PCR System (Thermo Fisher Scientific, Waltham, MA). *GAPDH* was used as an internal reference. The 2^–ΔΔCt^ method was used for relative expression analysis (12). All primer sequences were listed in Table 2.

**TABLE 2:** Sequences of primers for qRT-PCR.

**Gene name**	**Forward primer sequences (5′–3′)**	**Reverse primer sequences (5′–3′)**
*BIRC3*	TGGTGGACTCAGGTGTTGGG	CTGGCTTGAACTTGACG GATG
*NFKBIA*	CTGAAGAAGGAGCGGC TACTG	GCAGGTTGTTCTGGAAG TTGAG
*CXCL1*	CCCAAACCGAAGTCATAGCC	AAACACATTAGGCACAA TCCAGG
*ICAM1*	ACATCTGTGTCCCCCT CAAAAG	CGGGGTCTCTATGCCCAACA
*CCL2*	AAGCAGAAGTGGGTTCA GGATT	TCTTGGGTTGTGGAGTG AGTGT
*TNFAIP3*	ATGCCGCAAAGTTGGATGA	CTCGCTGTTTTCCTGCCATT
*JUNB*	AAAATGGAACAGCCCTT CTACC	GGTTTCAGGAGTTTGTAG TCGTGT
*GAPDH*	GGAGCGAGATCCCTCC AAAAT	GGCTGTTGTCATACTTCT CATGG

### CCK8 Assay for Cell Viability Analysis

Thirty thousand cells were planted in each well of a 96-well plate, and the culture medium was removed after 24 h. The cells were further cultured for 24 h by adding a culture medium containing lymph and/or drugs. For CCK8 assay, medium and CCK8 were mixed at a ratio of 10:1 and added into each well for a 4-h incubation. The absorbance at 450 nm was detected by Microplate Reader (SpectraMax^®^ M3, Molecular Devices, Sunnyvale, CA).

### Western Blot

Protein samples were extracted from HUVECs with RIPA lysis buffer and sonicated briefly. Equal amounts (30 μg) of protein from each group were separated on a 15% SDS–polyacrylamide gel (SDS-PAGE), and then transferred onto PVDF membranes (Millipore). The membranes were blocked with 5% milk in TBS-T for 1 h at room temperature and then incubated with primary antibody against CCL2 (66272-1-lg, Proteintech Group) at 4°C overnight. After three washes in TBS-T, the membranes were incubated with goat anti-mouse IgG HRP secondary antibody (Applygen) for 1 h at room temperature. The ImageQuant LAS 4000 imager was used for the detection of the protein expression, and the signal density was analyzed by Quantity One software.

### ELISA Analysis

The CCL2 concentration in NML and PHSML was measured by rat-specific ELISA kits (CD35498, Wuhan Chundu Biotechnology Co., Ltd, Wuhan, China) in accordance with the manufacturer’s protocols after manufacturing standard curves. The optical density at 450 nm was read using the Microplate Reader.

### Statistical Analysis

In this study, data of HUVEC viability and CCL2 concentration were presented as mean ± standard deviation (SD), and the data of mRNA expressions were presented as mean ± standard error (SE). Statistical analysis was performed using SPSS 16.0 (Polar Engineering and Consulting Inc., Chicago, IL, United States). One-way analysis of variance was used between multiple groups, and a Student’s *t*-test was used between two groups. *P* < 0.05 was considered to indicate a statistically significant difference.

## Results

### PHSML Treatment Induced Significant Gene Expression Change in HUVECs

**FIGURE 1:**
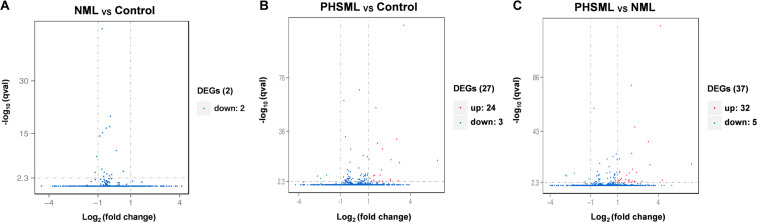
Volcano map of differentially expressed genes. HUVECs are treated by physiological saline (Control), normal mesenteric lymph (NML) and post-hemorrhagic shock mesenteric lymph (PHSML), respectively. **(A)** Distribution of differentially expressed genes between NML and Control groups. **(B)** Distribution of differentially expressed genes between PHSML and Control groups. **(C)** Distribution of differentially expressed genes between PHSML and NML groups. Green: down-regulated; Red: up-regulated, Blue: unchanged.

**FIGURE 2:**
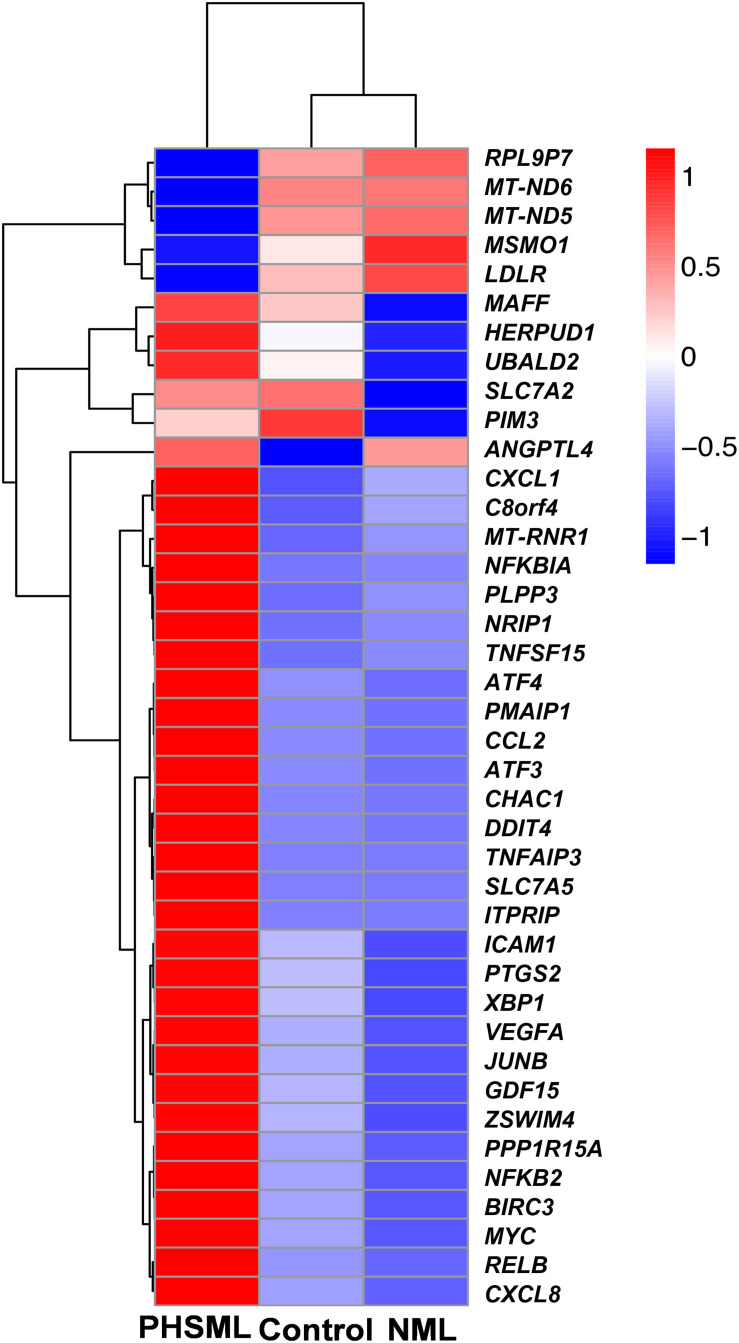
Heatmap of differentially expressed genes in various experimental groups. HUVECs are treated by physiological saline (Control), normal mesenteric lymph (NML), and post-hemorrhagic shock mesenteric lymph (PHSML), respectively.

Between NML and PBS groups, there were only two differentially expressed genes, which were slightly down-regulated in the NML group (Figure 1A). Between the PHSML and PBS groups, 24 genes were up-regulated, with 3 genes down-regulated in the PHSML group (Figure 1B). Compared with the NML group, 32 genes were up-regulated and 5 genes were down-regulated in the PHSML group (Figure 1C). The differentially expressed genes are listed in Tables 3–5. These results indicated that NML had little effect on HUVECs, but PHSML induced a significant change in gene expression pattern. According to the FPKM value of differentially expressed genes, hierarchical clustering analysis further proved the above results from the Tables 3–5 (Figure 2), which show the consistency of differentially expressed genes. NML or PHSML can simulate the physiological or pathological processes of vascular endothelium after lymph return into the circulation system, meaning we need to further analyze the differences between the PHSML and NML groups in the following analysis.

**TABLE 3:** Differentially expressed genes in NML compared to control.

**Gene ID**	**Gene name**	**log2FC**	**Qvalue**	**Change (up/down)**
ENSG00000003989	*SLC7A2*	−1.1712	0.0001267	down
ENSG00000198355	*PIM3*	−1.0755	3.63E-09	down

*HUVECs are treated by PBS (Control) and normal mesenteric lymph (NML), respectively.*

**TABLE 4:** Differentially expressed genes in PHSML compared to control.

**Gene ID**	**Gene name**	**log2FC**	***Q*-value**	**Change (up/down)**
ENSG00000023445	*BIRC3*	3.1139	0.0012607	Up
ENSG00000073756	*PTGS2*	1.6277	3.33E-30	Up
ENSG00000077150	*NFKB2*	1.4384	0.0045636	Up
ENSG00000100906	*NFKBIA*	1.91	2.66E-11	Up
ENSG00000103257	*SLC7A5*	1.9876	5.54E-26	Up
ENSG00000104856	*RELB*	1.7975	0.0030862	Up
ENSG00000108691	*CCL2*	3.0081	3.61E-33	Up
ENSG00000118503	*TNFAIP3*	1.9913	0.0041682	Up
ENSG00000128272	*ATF4*	1.0227	6.79E-19	Up
ENSG00000128965	*CHAC1*	5.931	5.19E-18	Up
ENSG00000130513	*GDF15*	1.5255	2.63E-55	Up
ENSG00000132003	*ZSWIM4*	1.1879	0.0032809	Up
ENSG00000141682	*PMAIP1*	1.739	0.004622	Up
ENSG00000162407	*PLPP3*	1.0985	2.75E-05	Up
ENSG00000162772	*ATF3*	2.5669	0.0013411	Up
ENSG00000163739	*CXCL1*	2.6025	1.62E-18	Up
ENSG00000167772	*ANGPTL4*	1.3371	0.00034481	Up
ENSG00000168209	*DDIT4*	3.2192	2.24E-16	Up
ENSG00000169429	*CXCL8*	3.5174	6.54E-114	Up
ENSG00000171223	*JUNB*	1.8258	1.38E-07	Up
ENSG00000176907	*C8orf4*	1.4414	1.38E-07	Up
ENSG00000180530	*NRIP1*	1.2024	0.0039945	Up
ENSG00000181634	*TNFSF15*	1.3505	4.41E-07	Up
ENSG00000198695	*MT-ND6*	−2.6541	6.40E-07	Down
ENSG00000198786	*MT-ND5*	−2.0139	1.38E-07	Down
ENSG00000211459	*MT-RNR1*	2.5518	0.00015293	Up
ENSG00000238103	*RPL9P7*	−2.4463	3.29E-05	Down

*HUVECs are treated by PBS (Control) and post-hemorrhagic shock mesenteric lymph (PHSML), respectively.*

**TABLE 5:** Differentially expressed genes in PHSML compared to NML.

**Gene ID**	**Gene name**	**log2FC**	***Q*-value**	**Change (up/down)**
ENSG00000003989	*SLC7A2*	1.1227	0.00018307	Up
ENSG00000023445	*BIRC3*	4.3015	8.06E-05	Up
ENSG00000051108	*HERPUD1*	1.8409	3.01E-05	Up
ENSG00000052802	*MSMO1*	−1.4188	0.00069419	Down
ENSG00000073756	*PTGS2*	2.264	3.64E-47	Up
ENSG00000077150	*NFKB2*	1.7928	0.00022588	Up
ENSG00000087074	*PPP1R15A*	1.163	5.05E-12	Up
ENSG00000090339	*ICAM1*	2.2326	0.00060164	Up
ENSG00000100219	*XBP1*	1.0116	0.0028753	Up
ENSG00000100906	*NFKBIA*	1.8447	1.35E-10	Up
ENSG00000103257	*SLC7A5*	2.0321	3.37E-26	Up
ENSG00000104856	*RELB*	2.0716	0.00053765	Up
ENSG00000108691	*CCL2*	3.2663	1.30E-35	Up
ENSG00000112715	*VEGFA*	1.4808	0.0049268	Up
ENSG00000118503	*TNFAIP3*	2.0213	0.0045237	Up
ENSG00000128272	*ATF4*	1.1353	8.15E-22	Up
ENSG00000128965	*CHAC1*	6.463	9.34E-18	Up
ENSG00000130164	*LDLR*	−1.1272	7.91E-06	Down
ENSG00000130513	*GDF15*	2.0031	2.14E-80	Up
ENSG00000132003	*ZSWIM4*	1.584	2.69E-05	Up
ENSG00000136997	*MYC*	1.1282	7.34E-11	Up
ENSG00000141682	*PMAIP1*	1.8859	0.0021706	Up
ENSG00000148841	*ITPRIP*	1.0093	0.0017979	Up
ENSG00000162407	*PLPP3*	1.0305	0.00016526	Up
ENSG00000162772	*ATF3*	2.8	0.00053765	Up
ENSG00000163739	*CXCL1*	2.0819	1.22E-13	Up
ENSG00000168209	*DDIT4*	3.378	5.71E-17	Up
ENSG00000169429	*CXCL8*	4.1646	1.31E-127	Up
ENSG00000171223	*JUNB*	2.3782	5.27E-11	Up
ENSG00000176907	*C8orf4*	1.2128	1.88E-05	Up
ENSG00000181634	*TNFSF15*	1.2795	2.88E-06	Up
ENSG00000185022	*MAFF*	1.6752	7.25E-09	Up
ENSG00000185262	*UBALD2*	1.0101	2.69E-05	Up
ENSG00000198695	*MT-ND6*	−2.7707	2.80E-08	Down
ENSG00000198786	*MT-ND5*	−2.2116	1.73E-10	Down
ENSG00000211459	*MT-RNR1*	2.2732	0.00080453	Up
ENSG00000238103	*RPL9P7*	−2.8438	6.85E-09	Down

*HUVECs are treated by normal mesenteric lymph (NML) and post-hemorrhagic shock mesenteric lymph (PHSML), respectively.*

### Bioinformatic Analysis of Differentially Expressed Genes

**FIGURE 3:**
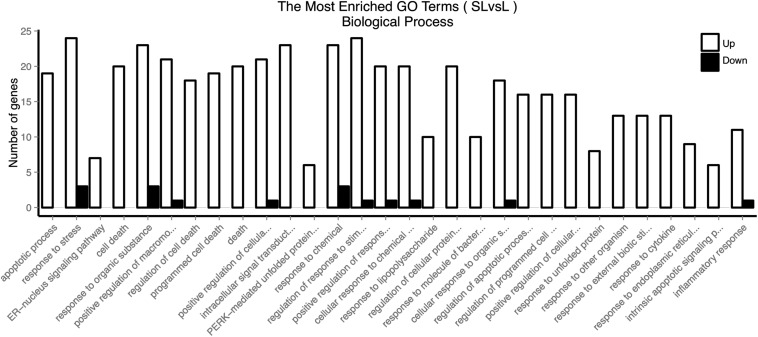
GO enriched biological processes of differentially expressed genes in HUVECs treated with NML and PHSML, respectively.

Firstly, we used GOSeq software for GO enrichment analysis (11). The differentially expressed genes were enriched in 2378 biological processes. We selected 30 GO terms with the most significant enrichment for further analysis (Figure 3). These differentially expressed genes were mainly involved in cell death, response to stress, endoplasmic reticulum nuclear signaling pathway, organic reaction, positive regulation of macromolecule metabolism, adhesion signal, and inflammatory response. It was evident that PHSML may affect the survival of HUVECs.

**FIGURE 4:**
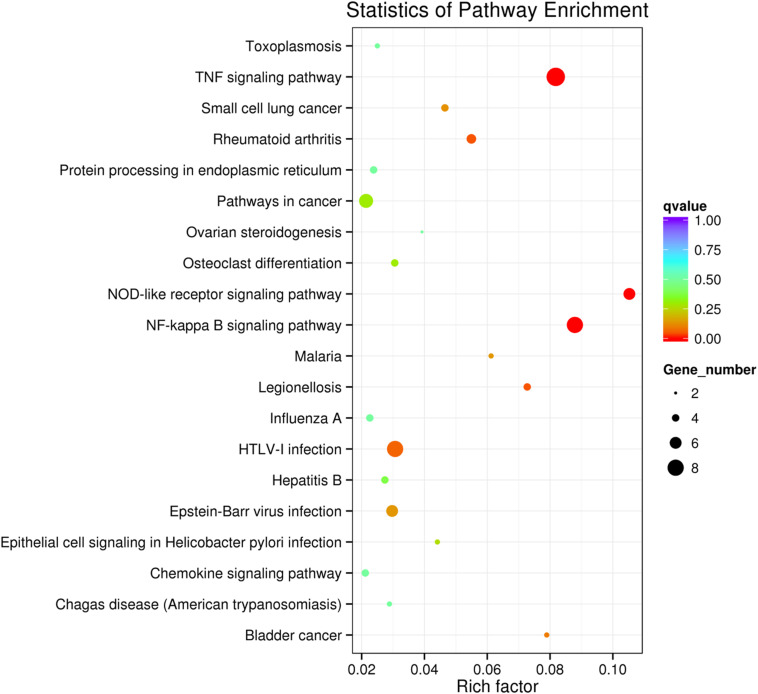
Scatter plot showing the enrichment of KEGG pathways (PHSML *vs* NML). HUVECs are treated by NML and PHSML, respectively. The size of dots represents the number of differentially expressed genes in this pathway. The point’s color represents the range of the *q*-value (the closer to zero, the more significant the enrichment is).

**FIGURE 5:**
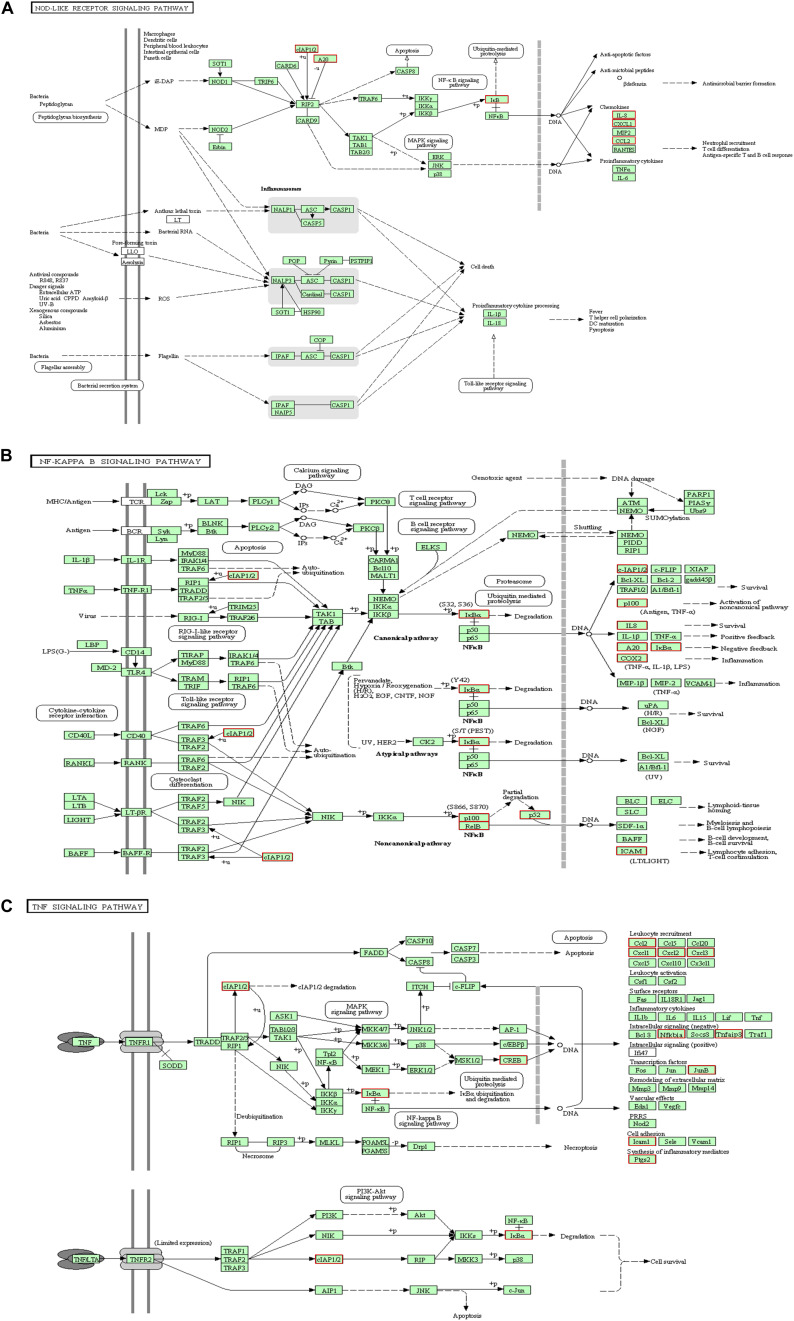
KEGG analysis for signaling pathways. The red box indicates that the up-regulated genes (PHSML vs NML). **(A)** NOD like receptor signaling pathway. **(B)** NF-κB signaling pathway. **(C)** TNF signaling pathway.

**FIGURE 6:**
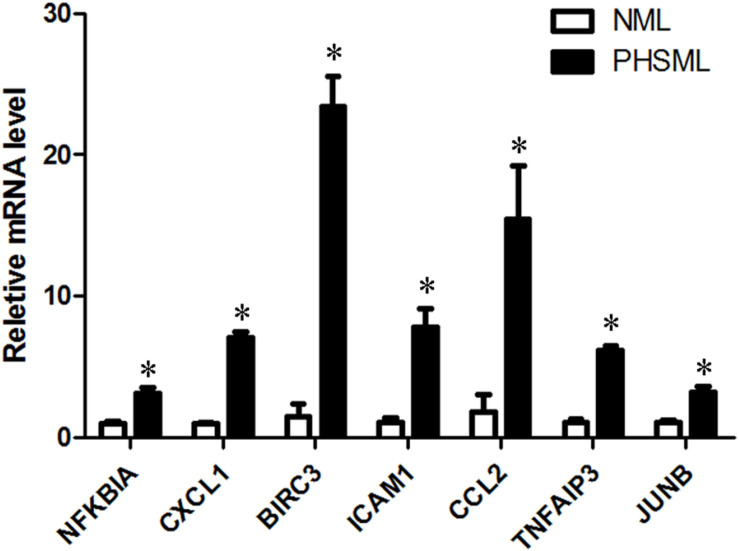
The verification of up-regulated genes (PHSML vs NML) by qRT-PCR. The data were shown as fold change, and expressed as mean ± SE (*n* = 3), ^∗^*P* < 0.05. All the original values of these seven genes in NML group were normalized to 1, respectively.

KEGG collects a large amount of signal pathway information, which can be used to analyze the network of intermolecular interactions (13). The differentially expressed genes between PHSML and NML enriched 105 signaling pathways in the KEGG database. Among the top 20 pathways, the nucleotide-binding oligomerization domain NOD-like receptor signaling pathway, nuclear factor (NF)-κB signaling pathway, and Tumor necrosis factor (TNF) signaling pathway each showed the highest degree of enrichment (Figure 4) respectively. Six differentially expressed genes (BIRC3, TNFAIP3, NFKBIA, CXCL8, CXCL1, and CCL2) were enriched into NOD-like receptor signaling pathway (Figure 5A). Eight differentially expressed genes (BIRC3, NFKBIA, NFKB2, RELB, CXCL8, TNFAIP3, PTGS2, and ICAM1) were enriched into the NF-κB signaling pathway (Figure 5B). Nine differentially expressed genes (BIRC3, NFKBIA, ATF4, CCL2, CXCL1, TNFAIP3, JUNB, ICAM1, and PTGS2) were enriched into the TNF signaling pathway (Figure 5C). Among them, cIAP1 and cIAP2 as regulators of innate immune signaling in mammals, induce robust RIP2 ubiquitination. Moreover, cIAP1 and cIAP2 function as K63 ubiquitin ligases for RIP1, which allows RIP1 to activate the prosurvival NF-kB pathway while preventing it from binding to caspase-8 and inducing apoptosis (14). TNFAIP3 (A20) leads to the ubiquitination of TRAF6 and the deubiquitination of RIP to inhibit the activation of the NF-κB signaling pathway that is mediated by NOD1 and NOD2 (15). Moreover, the genes for the chemokines (CXCL8, CXCL1, and CCL2) have inherent antimicrobial properties. These chemokines play an important role in neutrophil attraction and have been implicated in the prevention of infection in the lung epithelium. Interestingly, these three signaling pathways and the differentially expressed genes are mostly related to inflammation. Thus, excessive inflammation during hemorrhagic shock might further cause tissue damage. To validate the above analysis, seven genes (BIRC3, NFKBIA, CXCL1, ICAM1, CCL2, TNFAIP3, and JUNB) were randomly selected for quantitative real-time polymerase chain reaction (qRT-PCR) verification. The qRT-PCR results demonstrated that our sequencing data were reliable (Figure 6).

### Bindarit Alleviated the Damage of PHSML to HUVECs

The above results of RNA-seq and qRT-PCR showed that CCL2 was significantly increased after PHSML treatment, indicating it may play an important role in PHSML stimulated HUVEC injury. Therefore, CCL2 production inhibitor Bindarit might attenuate the cell injury by PHSML, and detected the effects of Bindarit on cell morphology and viability.

**FIGURE 7:**
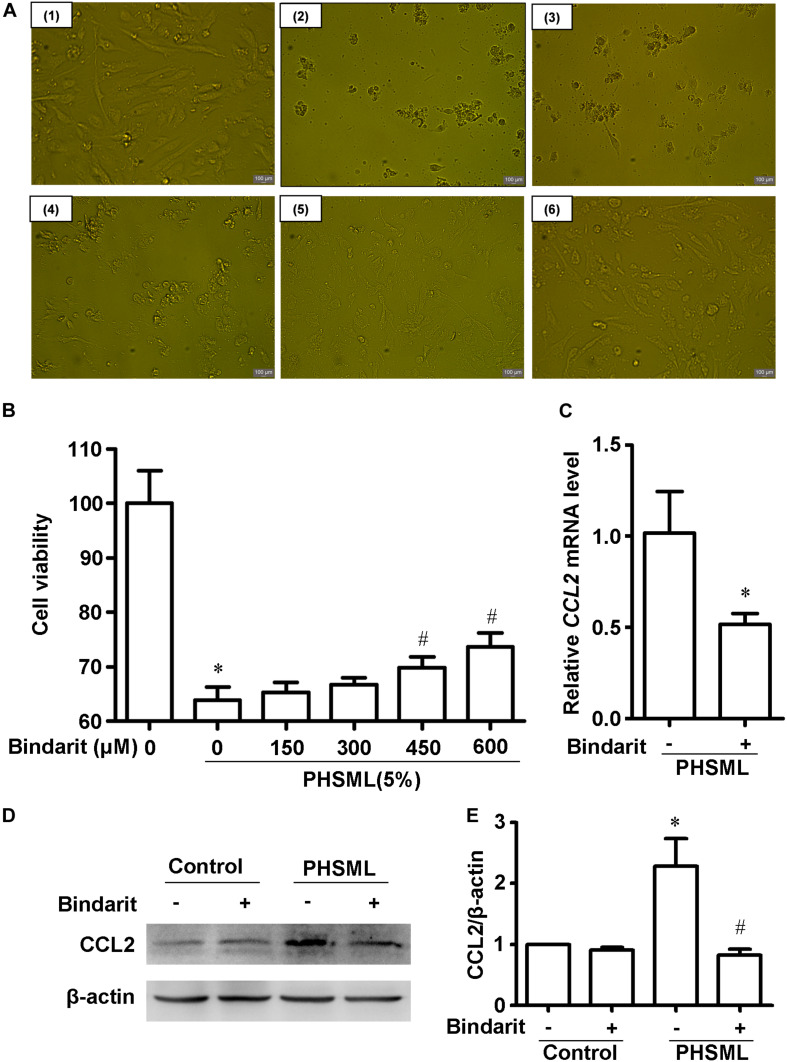
Effect of Bindarit on PHSML-induced HUVECs injury. **(A)** The effect of Bindarit on the morphology of HUVECs treated by PHSML for 24 h. **(1)** Control; **(2)** 5% PHSML; **(3)** 5% PHSML + 150 μM Bindarit; **(4)** 5% PHSML + 300 μM Bindarit; **(5)** 5% PHSML + 450 μM Bindarit; **(6)** 5% PHSML + 600 μM Bindarit. **(B)** The effect of Bindarit on the viability of HUVECs treated by PHSML for 24 h. The data is expressed as mean ± SD (*n* = 10). ^∗^*P* < 0.05, compared with the control group; ^#^*P* < 0.05, compared with the PHSML group. **(C)** The effect of Bindarit (300 μM) on *CCL2* mRNA expression in HUVECs treated with PHSML. The expression was calculated by 2^–ΔΔCt^ method. The data is expressed as mean ± SE (*n* = 3), ^∗^*P* < 0.05, compared with PHSML group. **(D)** Western blot analysis showed decreased expression of CCL2 protein after Bindarit treatment. **(E)** Quantitative analysis of Western blot results from panel **(D)**. The data is expressed as mean ± SE (*n* = 3). ^∗^*P* < 0.05, compared with the control group; ^#^*P* < 0.05, compared with the PHSML group.

Compared with control cells, PHSML treated cells shrank obviously after 24-h incubation, with the appearance of cell debris (Figure 7A). Thus, PHSML treatment for 24 h induced the morphological injury and decreased viability of HUVECs significantly. We found the morphology of HUVECs was partially improved with the increase of Bindarit concentration (Figure 7A). Meanwhile, the results were further confirmed using a CCK8 assay to detect cell viability. When the concentration of Bindarit reached 450 and 600 μM, cell viability was significantly increased (Figure 7B). Quantitative PCR showed the mRNA expression of CCL2 was significantly decreased by Bindarit treatment (Figure 7C). Furthermore, Western blot results confirmed that the PHSML-induced CCL2 expression was inhibited by Bindarit, indicating the inhibitor was effective (Figures 7D,E). Therefore, Bindarit, an inhibitor of CCL2 production, could alleviate the damage of HUVECs by PHSML.

### CCL2 Concentration in Mesenteric Lymph

The result from the ELISA assay showed that there were no statistical differences in CCL2 level between the PHSML (84.65 ± 5.16 ng/ml) and NML (81.42 ± 6.83 ng/ml).

## Discussion

Hemorrhagic shock increases vascular permeability and induces organ damage, which is related to mesenteric lymph return to the blood circulation system (16). Plasma constituents and blood cells can pass through the vascular endothelial barrier through cellular and paracellular pathways (17). It has been shown that PHSML treatment increased the permeability of HUVECs when the concentration of shock lymph reached 10% in a culture medium, while shock venous serum had no effects (4, 5). In order to dissect the molecular mechanism of PHSML injury on endothelial cells, we performed RNA-seq on HUVECs incubated with 10% PBS, 10% NML, and 10% PHSML, respectively. Furthermore, we verified the mediating role of CCL2, a representative gene among differentially expressed genes, in the impairment of HUVECs induced by PHSML.

According to the data of the RNA-seq, PHSML-treated HUVECs exhibited significant changes in 37 gene expression, while expressed genes in PBS and NML group showed a similar pattern with no changes. We found that these differentially expressed genes are highly enriched in the NOD-like receptor signaling pathway, the NF-κB signaling pathway, and the TNF signaling pathway in the KEGG database. These pathways are closely related to inflammation and immune response, and crosstalk among the three pathways exists. NOD-like receptors are cytoplasmic pattern recognition receptors that contribute to the activation of inflammatory caspases and have been shown to link to inflammatory responses, immune defense, and tissue injury in models of hemorrhagic shock (18–20). Moreover, NOD-like receptors can activate the NF-κB signaling pathway when they recognize danger-related signaling molecules and pathogen-related signaling molecules (21, 22), following hemorrhagic shock (20). TNF is a multifunctional cytokine family that plays an important role in immunity, inflammation, cell proliferation, and differentiation (23). It has been demonstrated that TNF-α is a major cause of lung endothelial injury after lung ischemia-reperfusion (21, 24), which is consistent with the lung injury induced by hemorrhagic shock (25). Furthermore, TNF-α is an important mediator of endothelial dysfunction through the activation of NF-κB and upregulation of NF-κB components (26, 27). Therefore, both the NOD-like receptor signaling pathway and the TNF signaling pathway are involved in NF-κB pathway activation. When cells are in a state of stress and injury, NF-κB, as a coordinator, participates in the expression of various cytokines and adhesion molecules and enhances the immune response. Importantly, the NOD-like receptor signaling pathway (28, 29), the NF-κB signaling pathway (30–32), and TNF signaling pathway (33–35) are all involved in endothelial cell injury. Thus, the inter-related three pathways may form a complicated network that synergistically induces inflammatory injury.

PHSML return plays an important role in the pathogenesis of hemorrhagic shock-induced orange injuries and inflammation. Previous studies have demonstrated that PHSML treatment causes NF-κB activation and I-κB degradation and induces human pulmonary endothelial cells damage (36), intravenous injection of PHSML significantly increased the levels of TNF-α in the plasma of the lung, kidney, etc. (37). The activation of the NOD-like receptor pyrin domain containing-3 (NLRP3) inflammasome is involved in hemorrhagic shock-induced acute lung injury and lung endothelial cells damage through inflammatory response and cell pyroptosis (19, 38, 39). All these factors are related to PHSML effects that need to be determined in future research. Previous studies have demonstrated that NLRP3 siRNA transfection abolished palmitate-induced IL-1β release in HUVECs (40). Blockade of NF-κB activation with a specific inhibitor, BAY 11-7085, partially overthrown gamma radiation-induced cellular hyper-permeability, decreased viability, and excessive inflammation in HUVECs (41). Inhibition of TNF-α with adalimumab also attenuated inflammatory responses in long-term HUVEC cultures (42). These results have confirmed that all NOD-like receptors, NF-κB, and TNF pathways participate in the HUVECs damage induced by various pathogenic factors.

Interestingly, in three groups of differentially expressed genes related to the aforementioned three pathways, several genes that can inhibit inflammation were also altered after PHSML treatment. BIRC3 is a member of the inhibitor of apoptosis family and can bind to TNF receptors TRAF1 and TRAF2 (43). TNFAIP3 is a zinc-containing protein. When some innate immune receptors, such as NOD-like receptors and TNFR, activate the NF-κB signaling pathway, the OTU domain of TNFAIP3 can induce deubiquitination of many related factors such as TRAF6 of the NF-κB signaling pathway, and inhibit the inflammatory response. It has been reported that after renal ischemia-reperfusion, TNFAIP3 can alleviate renal tissue injury by inhibiting the NF-κB signaling pathway and anti-inflammatory reaction (44). To date there are no reports on the role of TNFAIP3 in the inflammatory response after hemorrhagic shock. It can be speculated that the increased TNFAIP3 might be a compensatory response in HUVECs following PHSML treatment. Similarly, NFKBIA, which is considered an anti-inflammatory protein because it inhibits NF-κB (45), was also upregulated. Therefore, further investigation of these anti-inflammatory genes and how they are altered during PHSML treatment warrants further attention in future studies.

Chemokines are small inflammatory proteins that can induce leukocytes to express integrins, recruit leukocytes, and produce superoxide anions. These functions are mainly the first defense against microbial invasion and tissue damage (46). However, when the homeostasis of these molecules is unbalanced and maintains a high level, numerous inflammatory cells will be recruited to the injured site, which expands the inflammatory response and causes more severe tissue damage. According to the location of N-terminal cysteine residues, these factors are divided into four categories: C, CC, CXC, and CX3C (47). In our study, CXCL1, CXCL8, and CCL2 are significantly increased after PHSML treatment. These chemokines play an important role in the development of inflammation. After hepatic hemorrhage and reperfusion, CXCL1 expression was increased, and a large number of neutrophils were recruited to the liver, resulting in aggravation of liver injury (48). Repertaxin, an inhibitor of the CXCL8 receptor, alleviates this inflammatory response, and intestinal ischemia-reperfusion injury (49). After hepatic hemorrhage and reperfusion, the inflammatory response of CCL2-deficient mice was inhibited and the liver was protected (50). In light of these results, it is reasonable to conceive that PHSML may cause tissue damage by inducing pro-inflammatory chemokines of an endothelial cell.

As a pro-inflammatory chemokine, CCL2 is regulated by NF-kB in human aortic endothelial cells (51). Our results of RNA-seq have shown that CCL2 is upregulated by PHSML in HUVECs. Since PHSML-treated HUVECs showed enriched NF-kB signaling genes, CCL2 may be conservatively regulated by NF-κB in endothelial cells. Furthermore, we found that Bindarit, an inhibitor of CCL2 production (52), could protect HUVECs in survival activity and cytomorphology. Therefore, targeting differentially expressed genes can alleviate the damage of PHSML on HUVECs.

PHSML is cytotoxic to endothelial cells, including increased HUVEC permeability and decreased cell viability (53). In our study, we further identified three inflammatory pathways using RNA-seq and found that CCL2 participated in PHSML-induced endothelial cell injury. This result suggests a new strategy for the treatment of PHSML-induced organ injury by targeting the CCL2. However, the role of CCL2 on the secretory or barrier function of endothelial cells treated by PHSML needs to be investigated in the future.

In the present study, to clarify whether CCL2 in PHSML causes HUVECs damage, we performed an animal experiment to collect the mesenteric lymph and tested the CCL2 levels in NML and PHSML using the ELISA method. The result showed that there was no difference in the levels of CCL2 between NML and PHSML, suggesting that the HUVECs damage was not induced by CCL2 payload in PHSML. The component in PHSML that induces cellular injury requires further investigation. It should be noted that many scholars have determined the biologically active component analysis in PHSML that leads to cell damage and shock deterioration using various experimental technologies, for the potential therapeutic interventions of hemorrhagic shock targeting PHSML. Although these studies have identified a number of components carried in PHSML, further studies should be done, targeting the active factors and toxic substances contained in PHSML, a long and hard process.

It should be pointed out that there were certain limitations in the current study. Firstly, the present study demonstrated the role of Bindarit treatment in reducing the expression of CCL2 and in enhancing the viability of HUVECS. However, the effects of Bindarit treatment on the expressions of primary signaling molecules in the NOD-like receptor, NF-κB, and TNF signaling pathways were not determined. Secondly, the present study did not observe the roles of CCL2 inhibitors in *in vivo* animals following hemorrhagic shock. These issues warrant further investigation and could provide fundamental information for expanding clinical practice and targeting CCL2 in the treatment of hemorrhagic shock in the future.

Taken together, the findings of the present study have demonstrated that the altered genes in HUVECs treated with PHSML were involved in many pathways, including the NOD-like receptor signaling pathway, NF-κB signaling pathway, and TNF signaling pathway, which were most enriched. These pathways and the differentially expressed genes are closely related to inflammation, while inhibition of pro-inflammatory CCL2 can effectively alleviate the damage of PHSML to HUVECs. These results indicate a novel molecular mechanism responsible for the adverse effect of PHSML on vascular endothelium, which may represent a potential target in the treatment of hemorrhagic shock.

## Data Availability Statement

The datasets presented in this study can be found in online repositories. The names of the repository/repositories and accession number(s) can be found at: https://www.ncbi.nlm.nih.gov/bioproject/PRJNA602776/.

## Ethics Statement

The animal study was reviewed and approved by Animal Ethics Committee of Hebei North University.

## Author Contributions

Z-GZ, C-YN, and L-HN conceived and designed the work. QW, Z-FC, DW, Z-AZ, and HZ acquired and analyzed data for the work. L-MZ, Y-XL, A-LK, MZ, and PW participated in the animal experiments. QW, Z-AZ, and Z-GZ wrote the manuscript. Z-GZ and C-YN revised the manuscript. All authors contributed to the article and approved the submitted version.

## Conflict of Interest

The authors declare that the research was conducted in the absence of any commercial or financial relationships that could be construed as a potential conflict of interest.
